# Information about COVID-19 among Selected Population of Eastern Nepal: A Descriptive Cross-sectional Study

**DOI:** 10.31729/jnma.5096

**Published:** 2020-10-31

**Authors:** Kumud Chapagain, Gajendra Prasad Rauniyar, Rais Pokharel, Abhishek Bhattarai

**Affiliations:** 1 Department of Clinical Pharmacology and Therapeutics, B.P. Koirala Institute of Health Sciences, Dharan, Nepal; 2 Department of Otorhinolaryngology, Purbanchal University College of Medical Sciences, Gothgaun, Morang, Nepal; 3 Purbanchal University School of Engineering and Technology, Morang, Nepal

**Keywords:** *COVID-19*, *eastern Nepal*, *information*

## Abstract

**Introduction:**

Rapid spread of COVID-19 has become a major concern worldwide. Strong adherence to preventive measures can help to break the chain of the spread of coronavirus. We conducted this study to find out the extent of information general people of Eastern Nepal have regarding COVID-19 and their attitude and practice towards preventing its spread.

**Methods:**

A descriptive cross-sectional online study was done among the people of Eastern Nepal on knowledge, attitude, and practice related to COVID-19 from May 1^st^ to May 15^th^ after obtaining ethical clearance from the ethical review board (ERB) (ref no. 319/2020 P). A 20 item survey instrument was adapted using WHO course materials on an emerging COVID-19. A convenience sample method was used. Data were collected and entered in Statistical Packages for Social Services version 11.5. Point estimate at 95% Confidence Interval was calculated along with frequency and proportion for binary data.

**Results:**

Among 1069 respondents, the correct answer on the COVID-19 related knowledge questionnaire was 958 (89.61%), 487 (93.11%) were health professionals, and 471 (86.26%) non-health professionals. Preventive measures were strictly followed by 1044 (97.66%) participants. A wrong perception about the disease was present in 390 (36.48%). Health ministry website 356 (33.30%) followed by news media 309 (29%) was the major source of information among the people.

**Conclusions:**

Knowledge regarding COVID-19 among people the selected population of eastern is satisfactory which was similar to other studies done. However, people still have misperceptions regarding the disease and do not strictly follow the preventive measures.

## INTRODUCTION

Coronavirus disease 2019 (COVID-19), a global pandemic,^1^ has put the entire world into perturbation. An emerging respiratory disease^2^ caused by a new strain of the virus, novel coronavirus, was first detected in December 2019 in Wuhan, China.^3,4^ With more than six million cases worldwide, more than 370,000 people have lost their lives to the virus.^5^

In Nepal, a total of 2300 cases have been confirmed as of June 3, 2020, affecting 64 of the 77 administrative districts.^7^ Although the nation is in lockdown since 23^rd^ March 2020, an exponential increase in COVID-19 positive cases has demanded urgent needs to understand people's cognizance towards this disease.

Since the major approach to breaking this chain of spread is to stringently adhere to its preventive measures; this study was done to find out the extent of information general people of Eastern Nepal have regarding COVID-19, as well as their attitude and practice related to it.

## METHODS

An online descriptive cross-sectional study was conducted from 1^st^ May 2020 to 15^th^ May 2020 during the lockdown period of Nepal. Ethical approval was obtained from the ethical review board of the Nepal Health Research Council (319/2020 P). Depending on the author's networks with people, a preformed an online questionnaire was sent via email, social media, Facebook, Whatsapp, Twitter, etc. Nepalese residents aged 16 years and more, who understood the content of the questionnaire and consented to participate in the study, were informed about the objectives of the study. Declaration of anonymity and confidentiality were made and informed consent was obtained.

A 20 item survey instrument was developed using WHO course materials on emerging COVID-19.^8^ It was finalized after pretesting online on 30 randomly selected individuals from social media. The questionnaire mainly focused on demographics and knowledge, attitude, and practice related to COVID-19. Demographic variables included age, gender, occupation, and area of residence. Among 20 questions, 8 questions were related to knowledge, 6 questions related to attitude, and 6 questions related to practice.

Sample size was calculated using the following formula,

$$\begin{array}{ll} \text{n} & \text{= }{\text{Z}}^{\text{2}}\times \text{p}\times \text{q}/{\text{e}}^{\text{2}}\\ & \text{= }{\left(1.96\right)}^{\text{2}}\times (0.5)\times (0.5)/{\left(0.03\right)}^{\text{2}}\\ & \text{= }1068 \end{array}$$

Where,

n = sample size
Z = 1.96 at 95% Confidence Interval
p = population proportion, 50%
q = 1-p
e = margin of error, (3%)

A total of 4000 were invited to participate in the survey. However, based on the sample size calculation, the responses from the first 1069 participants were used for data analysis. Microsoft Excel was used for data entry and Statistical Package for Social Sciences version 11.5 was used for the analysis of data. Descriptive statistics such as frequency, mean, standard deviation, and percent were calculated to describe the characteristics of the sample and cross-tabulation for the distribution of knowledge, attitude, and practices of participants towards COVID-19. Point estimate at 95% Confidence Interval was calculated along with frequency and proportion for binary data.

## RESULTS

Out of 1069 respondents, the correct answer to the questions on the COVID-19 knowledge questionnaire was 958 (89.61%), among the health professionals was 487 (93.11%) and among non-health professionals was 471 (86.26%). The majority, i.e. 356 (33.30%) obtained reliable information about COVID-19 primarily from the health ministry website and news media 309 (29%). People exchanging information related to COVID-19 with family and friends were 232 (21.70%).

Virus as a cause of COVID-19 was the question with the highest correct answer 1045 (97.75%) and COVID-19 originated from bats had the least correct answer 712 (66.60%). A wrong perception that Nepalese are immune to COVID-19 was predominant on 390 (36.48%) and among them, 280 (71.79%) were the non-health professionals residing in rural areas 129 (63.86%) (Table 1).

**Table 1:** Knowledge about coronavirus disease 2019 (COVID-19) among the participants.

Question	Gender		Age		Residence		Occupation		Total correct response n (%)
	Male (n = 632)	Female (n = 437)	<30 yrs (n = 802)	>30 yrs (n = 267)	Urban (n = 867)	Rural (n = 202)	HPW (n = 523 )	NHPW (n = 546 )	
Coronavirus is the cause of COVID-19	623 (98.57)	422 (96.56)	792 (98.75)	253 (94.75)	860 (99.19)	185 (91.58)	517 (98.85)	528 (96.70)	1045 (97.75)
COVID-19 did not originate from bats	435 (68.82)	277 (63.38)	515 (64.21)	197 (73.78)	576 (66.43)	136 (67.32)	418 (79.92)	294 (53.84)	712 (66.60)
Fever, sore throat, headache, fatigue are symptoms of COVID-19	504 (79.74)	402 (91.99)	740 (92.26)	166 (62.17)	737 (85.00)	169 (83.66)	467 (89.29)	439 (80.40)	906 (84.75)
COVID-19 is transmitted through air, fecal-oral routes, and social contacts.	609 (96.36)	404 (92.44)	781 (97.38)	232 (86.89)	826 (95.27)	187 (92.57)	508 (97.13)	505 (92.49)	1013 (94.76)
Hand hygiene, covering nose and mouth while coughing, regular use of face mask helps in prevention of COVID-19 transmission	598 (94.62)	393 (89.93)	745 (92.89)	246 (92.13)	810 (93.42)	181 (89.60)	488 (93.30)	503 (92.12)	991 (92.70)
High-risk group people are prone to have severe symptoms of COVID-19	581 (91.93)	398 (91.07)	737 (91.89)	242 (90.63)	800 (92.27)	179 (88.61)	487 (93.11)	492 (90.10)	979 (91.58)
There is no role of antibiotics in treating COVID-19	585 (92.56)	394 (90.16)	739 (92.14)	240 (89.88)	798 (92.04)	181 (89.60)	496 (94.83)	483 (88.46)	979 (91.58)
Isolation and treatment of COVID-19 positive patient reduces spread of virus	628 (99.36)	406 (92.90)	784 (97.75)	250 (93.63)	842 (97.11)	192 (95.04)	512 (97.89)	522 (95.60)	1034 (96.72)

The overall practice of preventive measures was strictly followed by the majority, i.e. 1044 (97.66%) of the participants. However, only 550 (51.44%) thought there was adequate awareness regarding COVID-19 among the general public. The majority, i.e. 356 (33.30%) obtained reliable information about COVID-19 primarily from the health ministry website and news media 309 (29%). People exchanging information related to COVID-19 with family and friends were 232 (21.70%) (Table 2).

**Table 2:** Participants’ sources of information about coronavirus disease 2019 (COVID-19).

Response	Source of COVID-19 information			
	Health ministry websites n (%)	News media n (%)	Social media n (%)	Family and friends n (%)
Most often used	356 (33.30)	309 (28.90)	250 (23.33)	232 (21.70)
Often used	290 (27.12)	140 (13.09)	338 (31.61)	402 (37.60)
Sometimes used	183 (17.11)	302 (28.25)	300 (28.06)	304 (28.43)
Least used	240 (22.45)	318 (29.74)	181 (16.93)	131 (12.25)

Out of 1069 respondents, 632 (59.1%) were male, 523 (48.9%) were health professionals, 1011 (94.5%) below 50 years of age and 867 (81.10%) were from urban areas (Table 3).

**Table 3:** Table 3. Sociodemographic characteristics of the participants.

Variables	n (%)
Age	
16-29	691 (64.63)
30-49	320 (29.93)
50+	58 (5.42)
Gender	
Male	632 (59.12)
Female	437 (40.87)
Occupation	
Health professional	523 (48.92)
Non-health professional	546 (51.07)
Residence	
Urban	867 (81.10)
Rural	202 (18.89)

## DISCUSSION

Since the initial outbreak in Wuhan, China, in December 2019, COVID-19 has been having an outrageous effect worldwide.^3^ The best possible way to combat this situation lies in the hands of the general public who can well apprehend and strictly follow the preventive measures against COVID-19.

This study showed that 89.61% of participants correctly answered questions related to knowledge. A similar study conducted in China^9^ showed 70.2-98.6% and a study conducted in Uganda^10^ showed 69% had sufficient knowledge on the COVID-19. An acceptable level of correct answer by the participants in this study could be because the survey was conducted when the nation was already in the state of complete lockdown due to the COVID-19 pandemic and all the media, news channels were spreading various information and data regarding the COVID-19 pandemic. In this study participants mainly relied on the health ministry's websites (33.30%) and various news channels (28.90%) for gathering information regarding COVID-19 followed by social media (23.33%) and discussion with family and friends (21.70%). This finding goes contrary to the findings of the study conducted in the United Arab Emirates where 61% of the participants used social media as the major source of information.^11^ COVID-19 related updates disseminated by official government health authorities have positive insinuations for improving the awareness and knowledge of the general public and the health ministry, Government of Nepal has been continuously doing that every day. In this study, the range of correct answers provided by health professionals was better than non-professional groups (Table 2). This could be because medical personnel can comprehend medical knowledge better than any other professionals or people. Also, the males in this study had better knowledge than females whereas the majority of the females (91.99%) were better aware of the symptoms regarding COVID-19 than male participants (79.94%). In our study, almost 34% of the participants believed COVID-19 originated from bats. This may be due to the fact that COVID-19 was closely related to a wet market in China.^12^

In this study, the majority of the participants (88.02%) had a positive attitude towards lockdown preventing the spread of COVID-19, and 96.26% were well aware of the preventive measures. This could be because of the continuous efforts made by the Nepal government in strict regulation and implementation of the national lockdown and continuous advertisement by different news channels regarding the maintenance of hand hygiene and other preventive measures as a precaution. However, 63.51% of participants thought Nepalese were immune against coronavirus. This perception among many could be possible because at the time this study was conducted COVID-19 had already had its catastrophic effect in countries like Italy, the USA, and even India, but Nepal showed few positive cases only with minor mortality.

Along with the positive attitudes of the participants towards COVID-19, majority of the participants (97.10-98.40%) took precautions to prevent infection by COVID-19: avoiding crowded places, wearing face masks, following lockdown, and refraining from handshakes, etc. These stringent measures could be because of the ongoing national lockdown called by the national government and banning of public gatherings. However, this study still showed that 2.25% went to crowded places, 3% did not wear masks and 2.9% did not follow lockdown and social distancing. These risk behaviors were more attributed to participants residing in rural areas, and in participants less than 30 years of age. This could possibly be because young adults have risk-taking behavior^13^ and there is limited interference from the governing bodies like securities and policemen in the rural areas and people here are more of social nature and love to roam around chatting with neighborhood people.

Participation of the risk groups such as individuals with existing comorbid conditions, pregnancy, older people, and people from areas with limited internet access could not be included in the study. The data is selfreported depending on the respondent's honesty and ability to recall, this may be subject to recall bias. The convenience sampling method was used and a limited population was included, hence the findings cannot be generalized.

## CONCLUSIONS

Knowledge regarding COVID-19 among the people of the selected population of eastern Nepal is satisfactory which was similar to other studies done. Yet, a significant number of participants have misperceptions about being protected against COVID-19. The majority lack confidence and hesitate to lend a supporting hand towards the COVID-19 infected individual. As the threat of COVID-19 continues, greater efforts through educational campaigns and information dissemination through various means should be made to a wider population to improve their perceptions of the disease.
